# Migratory bats respond to artificial green light with positive phototaxis

**DOI:** 10.1371/journal.pone.0177748

**Published:** 2017-05-31

**Authors:** Christian C. Voigt, Manuel Roeleke, Lara Marggraf, Gunārs Pētersons, Silke L. Voigt-Heucke

**Affiliations:** 1 Department of Evolutionary Ecology, Leibniz Institute for Zoo and Wildlife Research, Berlin, Germany; 2 AG Verhaltensbiologie, Institute of Biology, Freie Universität Berlin, Takustr. 6, Berlin, Germany; 3 Institute for Biochemistry and Biology, University of Potsdam, Maulbeerallee 1, Potsdam, Germany; 4 Faculty of Veterinary Medicine, Latvia University of Agriculture, Jelgava, Latvia; Università degli Studi di Napoli Federico II, ITALY

## Abstract

Artificial light at night is spreading worldwide at unprecedented rates, exposing strictly nocturnal animals such as bats to a novel anthropogenic stressor. Previous studies about the effect of artificial light on bats focused almost exclusively on non-migratory species, yet migratory animals such as birds are known to be largely affected by light pollution. Thus, we conducted a field experiment to evaluate if bat migration is affected by artificial light at night. In late summer, we presented artificial green light of 520 nm wavelength to bats that were migrating south along the shoreline of the Baltic Sea. Using a light on-off treatment, we observed that the activity of *Pipistrellus nathusii* and *P*. *pygmaeus*, the two most abundant migratory species at our site, increased by more than 50% in the light-on compared to the light-off treatment. We observed an increased number of feeding buzzes during the light-on compared to the light-off treatment for *P*. *nathusii*. However, feeding activity was low in general and did not increase disproportionately during the light-on treatment in relation to the overall echolocation call activity of bats. Further, *P*. *nathusii* were attracted towards the green light at a distance of about 23 m, which is way beyond the echolocation detection range for insects of Nathusius’ bats. We therefore infer that migratory bats were not attracted to artificial green light because of high insect densities, but instead by positive phototaxis. We conclude that artificial light at night may potentially impact bat migration in a yet unrecognized way.

## Introduction

Artificial light at night is known to impair ecosystem functioning and to influence animal assemblages [1–5]. Yet artificial light at night is rapidly increasing worldwide, encroaching into previously dark habitats at unprecedented rates [3,4]. Strictly nocturnal animals such as bats are particularly vulnerable to artificial light (e.g. [6–9]). Previous studies highlighted that so called light-tolerant bat species, the majority of them belonging to the fast-flying bats, may hunt insects which are attracted to street lamps, particularly to those emitting energy in the short wavelength spectrum such as UV light [10–13]. Slow-flying species in contrast generally avoid artificial light, even in the presence of high food abundance [6–9, 14,15]. In all previous studies, researchers investigated the effect of artificial light at night on bats that either belonged to species with predominantly resident populations, i.e. with little or no seasonal migration, or to migratory species outside their migration period (reviewed in [15–17]). The single study focusing on the effects of artificial light on migrating bats was performed by Cryan and Brown who studied the response behaviour of North American *Lasiurus cinereus* towards the light of an offshore lighthouse [18]. They observed bats in the spotlight of the lighthouse, yet could not disentangle if bats were attracted by positive phototaxis or if they were hunting for insects. Here, we specifically asked if migratory bats are drawn towards artificial light by positive phototaxis, i.e. independent of the presence of insects lured by the artificial light.

It is well established that some nocturnal migrants, e.g. some bird species, fly towards and sometimes even collide with light sources at night. This may eventually lead to a higher mortality rate in migratory birds, either because disoriented individuals die when colliding with the light-carrying structure, e.g. lighthouses, tall buildings or towers; or because birds suffer from a negative energy balance when flying in the wrong direction [19–21]. The underlying causes for the attraction of migratory species towards light sources at night are still under debate, yet it seems likely that artificial light at night, particularly white light, might impair the use of celestial cues for visual orientation of birds and that red light might disrupt the magnetic orientation of birds (reviewed in [22]). Indeed experiments under laboratory conditions have established that the orientation of birds is most strongly affected by red and yellow light, i.e. under light of predominantly long wavelengths of the visible light spectrum [23–25]. Disorientation does not occur under light of predominantly short wavelengths such as blue and green light, probably because these wavelengths interact with the putative receptor molecule of the avian magnetic compass, i.e. cryptochrome1a (Cry1a; [25–29]. Consistent with these findings, Poot and colleagues observed that wild birds migrating across the North Sea get disoriented when flying towards red and white light, but birds showed no large response to blue and green light. This observation led to the recommendation to illuminate offshore platforms with green instead of red and white light to avoid attracting birds to these structures [30]. At the same time, the authors acknowledged that the effect of green artificial light on other organisms than birds still has to be investigated. Here, we tested whether migratory bats are attracted to green light.

Migratory bats may be particularly susceptible towards green artificial light because small-sized bats seem to be most sensitive towards light in the mid-range of the wavelength spectrum (520–540 nm; [31]). Indeed, most bats seem to be able to discern specific wavelengths, i.e. colours, since many Yangochiroptera (i.e. Vespertilioniformes) possess a small population (2–4%) of cones in the rod-dominated retinae [32–34]. Here we argue that artificial light at night might impair the orientation of migratory bats towards celestial cues, which are known to be used by bats for navigation [35]. If so, migratory bats may too suffer from disorientation during their annual journeys, for example when getting attracted to offshore platforms or buoys illuminated by green light. Offshore migration is rare in bats [36–38], mainly because migratory bats depend on hunting insects en route [39], which are rare or even absent over the sea. Thus, attraction of migratory bats towards anthropogenic structures illuminated by green light could be fatal when bats fly towards the sea in direction of such light sources.

In order to better understand the impact that artificial illumination at night has on bat migration, we tested whether migratory bats respond to green light at night when migrating in late summer (August, early September) from North-Eastern to South-Western Europe. We conducted an experiment at the Latvian coast of the Baltic Sea, where hundreds of thousands of bats pass by to reach their wintering sites in Central or South-western Europe [40]. In the centre of this migratory corridor, we placed a green-lighted surface on top of a pole which was illuminated by 520 nm laser light in a 10 min on and 10 min off scheme throughout the night. We then recorded the activity of bats using automated acoustical recorders when bats passed our experimental setup on their southwards migration. If migratory bats are attracted to artificial green light at night irrespective of whether it is due to increased insect densities during illumination or due to the light as such, we expected that the total number of echolocation calls should increase under the light-on compared to the light-off treatment. Further, we tested two hypotheses related to the question why bats are potentially attracted to the green light source, here focusing on the most abundant species *P*. *nathusii* [39,41]. If *P*. *nathusii* are hunting in the green light treatment for insects (attraction-by-insects hypothesis), we expected to see changes in the relative abundance of feeding buzzes (FB). During a feeding buzz, echolocation pulses are emitted in rapid succession with decreasing pulse intervals and decreasing peak frequencies, enabling bats to better detect an insect immediately before attack [42]. Specifically, we expected that the relative number of FBs in relation to the total number of echolocation calls should increase under the light-on compared to the light-off treatment, if bats hunt for insects attracted to the green light source [43]. Alternatively, if *P*. *nathusii* are attracted to artificial green light as such and not because of insects (attraction-by-artificial-light hypothesis), we predicted that the relative number of FB in relation to total echolocation calls per night should remain constant under both treatments.

## Material and methods

This study was conducted in 2015 near Pape Bird Ringing Station (PBRS; latitude 56.16306°, longitude 21.03964°) in Latvia during 14 nights between 18th of August and 3rd of September under the licence Nr. 10/2015 of the Latvian Nature Conservation Agency (issued on 02.04.2015) and the licence 2015-03-01 of the institutional animal care and ethics committee of the Leibniz Institute for Zoo and Wildlife Research. All methods were carried out in accordance with relevant guidelines and regulations, and all experimental protocols were approved by the institutional licensing committee. The study period included the peak migration season for *P*. *nathusii* (Nathusius’ bat), *P*. *pygmaeus* (Soprano bat) and *Nyctalus noctula* (Noctule bat) in Latvia [44,45]. All experiments were performed from dusk until dawn, except for one day when experiments stopped 4 hours earlier because of rain. At our experimental site, migrating bats usually fly consistently in north-south direction parallel to the coastal line [46], making it unlikely that the same individual is passing the experimental site repeatedly. We set up a line of three poles in an east-west orientation that was perpendicular to the heading direction of migratory bats (see S1 Fig). The two outer poles were placed each at 23 m distance to the central pole which carried the light source. The coastal pole was 100 m from the Baltic Sea. The central pole carried a north-facing white plastic board (0.4 m width x 4 m height) at 4.9 m height above ground. Along the 4 m length of the board, we installed a glass fibre which emitted a light of 520 nm through a laser source when turned on during the light-on treatment. During each of the 14 experimental nights, the laser light was switched on and off in 10 minute intervals; the dark period representing the control. At a height of about 6.2 m above ground, we equipped each pole with an omnidirectional ultrasonic microphone (Avisoft Electret FG from Knowles; Avisoft Bioacoustics, Berlin, Germany) pointing northwards to where the bats would appear. The ultrasonic microphones were attached to a computer. Using the software Avisoft-SASLab any bat that passed by the poles during their southward migration was automatically recorded from dusk till dawn.

We analyzed echolocation calls using the sound analysis program Avisoft-SASLab pro (Avisoft Bioacoustics, Berlin, Germany). Using this software, we first deleted all recordings that were solely triggered by insect noise or other noise interferences. In a second step, we applied a high-pass filter of 15 kHz to filter out any signals, such as insect noises or anthropogenic noises that hampered our ability to detect echolocation pulses. Then, we identified bat species, or species group, according to the species-specific pulse length of an echolocation call, the frequency curvature and maximum energy of it, using a combination of automated and subsequent individual inspection as analytical approach. To do so, we initially collected 25 to 30 frequently observed echolocation calls from an open-access library (www.ecoobs.de, [47]) for those species occurring at our experimental site, specifically: *P*. *nathusii*, *P*. *pygmaeus*, *Plecotus auritus*, *Myotis brandtii*, *M*. *nattereri*, *N*. *noctula*, *N*. *leisleri*, *Eptesicus serotinus*, *E*. *nilssonii*, *Vespertilio murinus*. We then generated spectrogram templates and used these for an automated identification using the software Avisoft SASLab pro. The program compares recorded echolocation calls with the deposited templates and tags identified calls based on visual resemblance with the species name in the recordings. Owing to the high variability and frequency overlaps of a species’ echolocation calls, the program did not identify silent calls and misidentified a large proportion of recorded echolocation calls produced by individuals of the nyctaloid group. Following Russo and Voigt [48], we visually double-checked and potentially corrected species tags in approximately 35.000 recordings. Due to their distinctiveness, the calls emitted by *P*. *nathusii* and *P*. *pygmaeus* were generally correctly identified. Based on their high similarity and the large intra-specific variation of echolocation call types from some species, we were not able to unambiguously identify the following species: *N*. *noctula*, *N*. *leisleri*, *E*. *serotinus*, *E*. *nilssonii*, *V*. *murinus*. Thus, we lumped together these species in the category nyctaloid.

To evaluate our predictions, we calculated the total number of echolocation calls recorded per night during the illuminated and dark period for each of the three microphones. Thus, each experimental day yielded two numbers for each pole: number of echolocation calls during the dark and number of echolocation calls during the illuminated phase. Since it is highly unlikely that bats pass by the experimental site repeatedly during the autumn migration period, we considered subsequent recordings to be independent [45,46]. Based on the 14 experimental nights, we then conducted statistical analysis for the most frequently observed species and species group, namely *P*. *nathusii*, *P*. *pygmaeus* and the nyctaloids. We performed for each of the aforementioned groups Wilcoxon tests, assuming an alpha value of 0.05. To test the first prediction (attractiveness of artificial light at night for migratory bats), we compared the cumulative number of echolocation calls recorded per night at the central pole when illuminated and when left dark. In order to evaluate if a potential attraction towards light was associated with feeding on insects that were lured to the light source, we visually identified feeding buzzes according to their stereotypic patterns [42]. We then tested if the number of FB in relation to the number of echolocation calls had been higher during the light-on than during the light-off treatment at the central pole. For this, we counted all FB emitted by *P*. *nathusii* and other bat species. Then, we tested for light-dependant variation of the relative number of FB from *P*. *nathusii* using a Wilcoxon test. We performed the analysis of acoustical data from the lateral poles separately, because we observed a higher activity of migratory bats at the coastal than at the inland pole; i.e. we compared the cumulative number of echolocation calls recorded per night at each of the lateral poles when illuminated and when left dark. Based on these two data sets, we used for each of the aforementioned groups Wilcoxon tests, assuming an alpha value of 0.05. Relevant data is made available in the supporting information.

## Results

**Fig 1:**
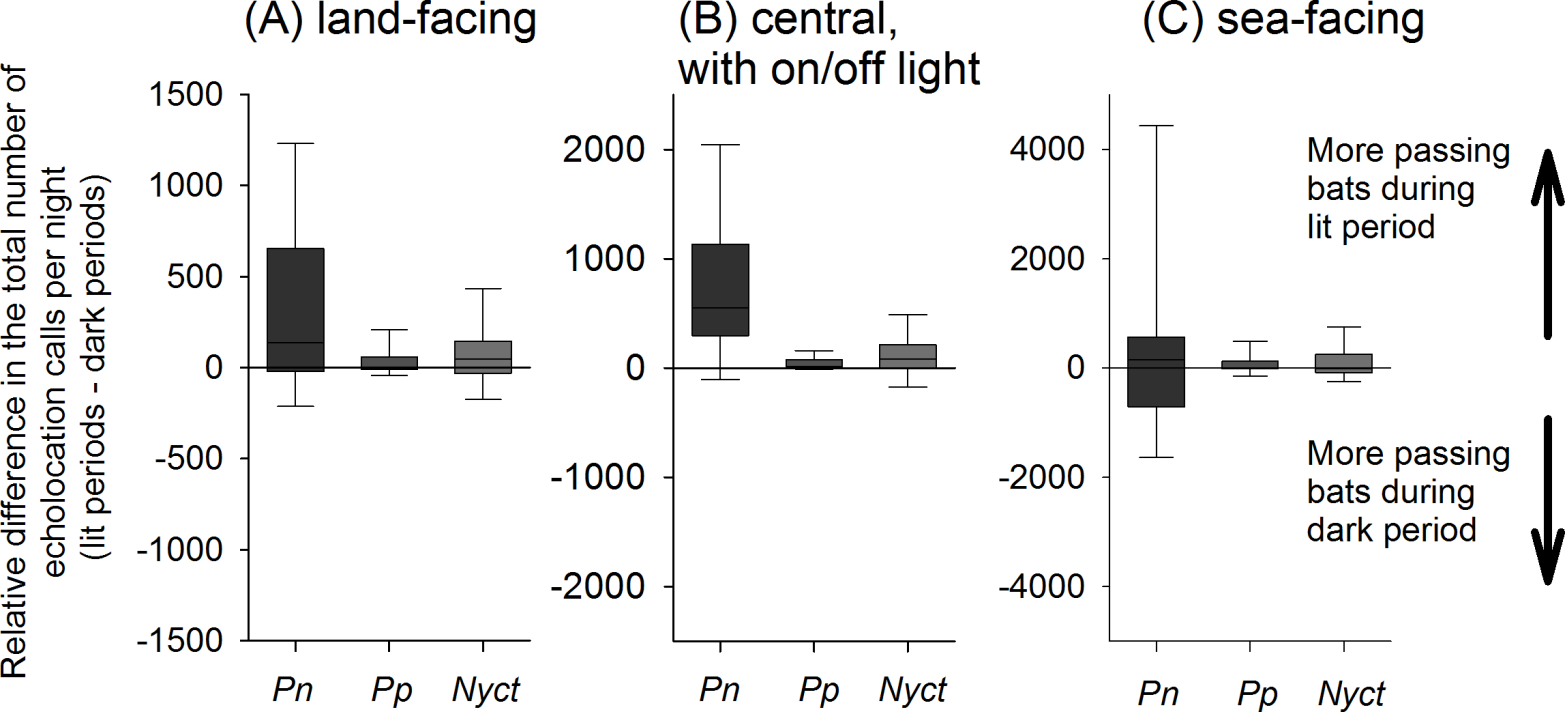
Difference in the cumulative number of echolocation calls recorded per night during the dark and light periods. Differences for *P*. *nathusii* (Pn), *P*. *pygmaeus* (Pp) and Nyctaloids (Nyct) at the landside pole (A), the central pole with the light on-off treatment (B) and the seaside pole (C). Data are depicted as boxplots with 25 and 75 percentiles as the border of the boxes and whiskers encompassing 5 and 95 percentiles.

We performed experiments during 14 nights, consisting of on average 28 ± 4.5 (mean ± one standard deviation) light-on light-off cycles per night (each 20 min in duration). We counted on average 21,448 ± 17,502 echolocation calls per night at all three poles taken together. We recorded an increasing number of echolocation calls from the inland to the coastal pole. At the inland pole, we counted 2,771 ± 2,039, at the central pole 4,656 ± 4,131 and at coastal pole 14,021 ± 12,192 echolocation calls per night. In total, we documented 163,575 calls during the light-on and 136,703 during the light-off period. We counted the largest number of echolocation calls for *P*. *nathusii* (248,402 out of 300,278 calls; 82.7% of all calls), followed by the nyctaloid group (39,063; 13.0%) and the Soprano bat *P*. *pygmaeus* (11,873; 4.0%). We rarely recorded echolocation calls of Brown long-eared bats *Plecotus auritus* (419; 0.1%) and *Myotis* species (521; 0.2%). Since these species were rare and are not migratory, we decided not to pursue further detailed analysis on them. Migratory Nathusius’ and Soprano bats were more frequently recorded at the central pole when the white board was illuminated than when it remained dark (*P*. *nathusii*: Wilcoxon, Z = 3.1, n = 14 pairs, p = 0.002; *P*. *pygmaeus*: Z = 2.5, n = 14 pairs, p = 0.011;Fig 1B, S1 Table). For the species included in the group of nyctaloids, we observed a trend for an attraction effect of bats towards the artificial light (Z = 1.92, n = 14 pairs, p = 0.055). The median percent increase in bat activity during the light-on period equalled 54% (minimum: -34%, maximum: 550%) for Nathusius’ bats and 47% (-100%; 594%) for Soprano bats compared with the light-off period. Further, when the central pole was illuminated, we observed an increased activity of Nathusius’ bats at the inland pole during the light-on period compared with the light-off period (Z = 2.17, p = 0.030) (Fig 1A, S1 Table). Yet, we did not observe an effect of the on-off light treatment for Soprano bats (Z = 1.02, p = 0.308) and the nyctaloids (Z = 1.36, p = 0.173; Fig 1A). At the coastal pole, we did not record any change in activity in response to the light on-off treatment (*P*. *nathusii*: Z = 0.157, P = 0.875; *P*. *pygmaeus*: Z = 0.94, P = 0.347; nyctaloid: Z = 0.80, p = 0.422; Fig 1C, S1 Table).

**Fig 2:**
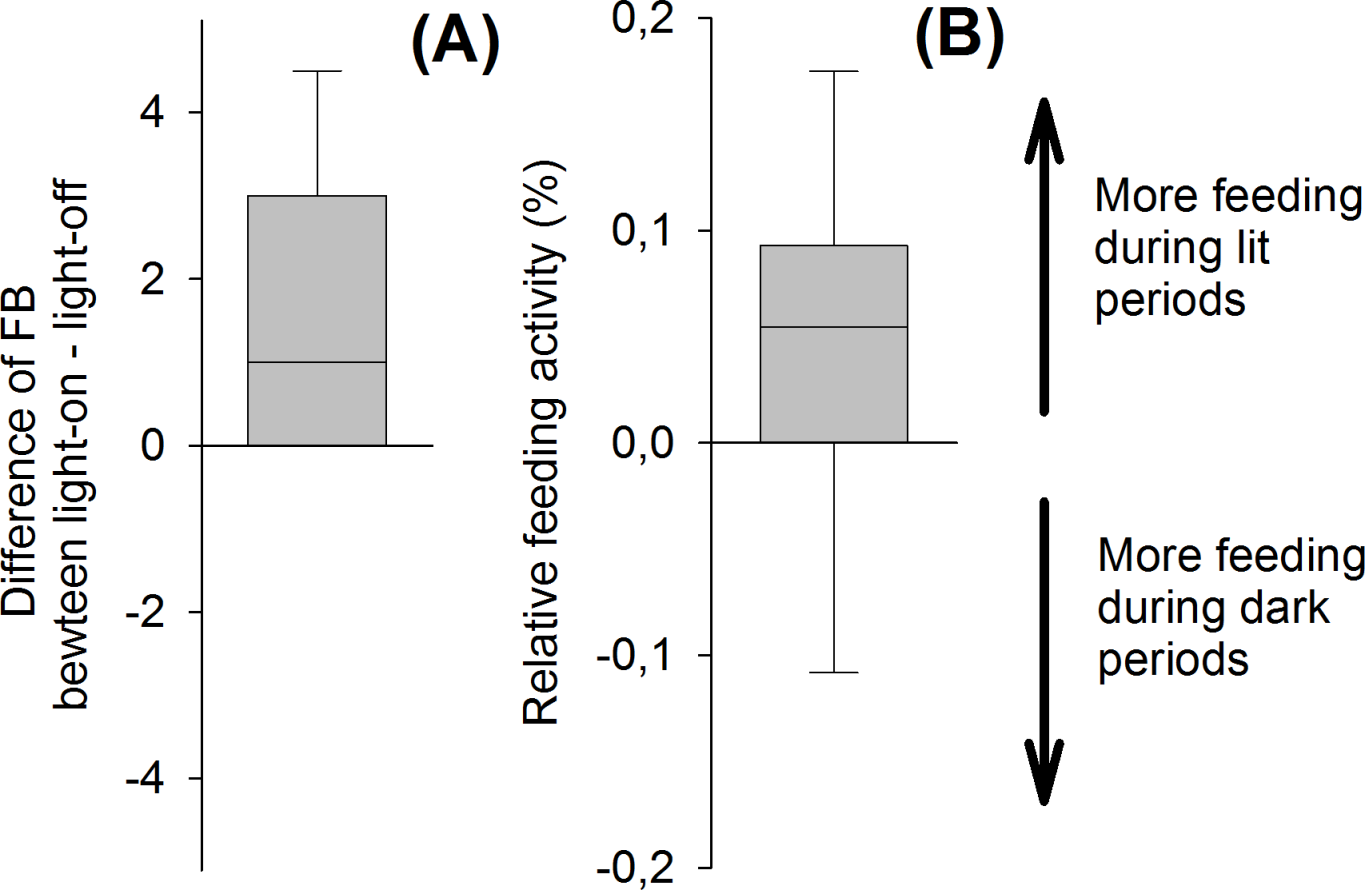
**Difference in the cumulative number of feeding buzzes (FB) recorded per night (A) and difference in the relative foraging activity per night (B) at the central pole for *P*. *nathusii***. Data are depicted as boxplots with 25 and 75 percentiles as the border of the boxes and whiskers encompassing 5 and 95 percentiles.

We recorded in total 237 FBs during our study period, of which 61.2% (n = 145) belonged to *P*. *nathusii* and 38.8% (n = 92) to other bat species. In total, we observed 10.4 ± 9.9 FBs per night for *P*. *nathusii* (range: 0–37; median 9) and 6.6 ± 7.9 FBs per night for other species at all three poles (range: 0–23, median 3). We analyzed if the overall and relative feeding activity of *P*. *nathusii* increased under the green light treatment. At the central pole, we observed 2.8 ± 2.6 FBs (range: 0–9 FB) per night for *P*. *nathusii*. On average, we recorded 1.5 ± 1.7 FBs (median = 1 FB) more under the light-on than under the light-off treatment (Z = 2.69, n = 14 pairs, p = 0.007, Fig 2A, S2 Table). To test whether this increase in feeding activity was disproportionately higher than the increased flight activity of *P*. *nathusii* during the light-on treatment, we calculated the relative proportion of FBs per echolocation call per night. We found a trend for a higher relative feeding activity of *P*. *nathusii* during the light-on compared to the light-off treatment (Z = 1.87, n = 14 pairs, P = 0.062) (Fig 2B). *Pipistrellus nathusii* emitted 0.0010 FB per echolocation call during the light-on treatment compared to 0.0006 FB per echolocation call during the light-off treatment (mean difference: 0.0004 ± 0.0009).

## Discussion

We tested if migratory bats, in particular *Pipistrellus nathusii*—the most abundant species at our study site -, respond to artificial green light at night during migration. We hypothesized that *P*. *nathusii* could be attracted to the artificial light because of two reasons, (1) because they hunt insects lured by the light (attraction-by-insect hypothesis) or (2) because they get disoriented when exposed to artificial light at night and thus head towards the light source (attraction-by-artificial-light hypothesis). Overall, we recorded a higher activity of migratory bats at the central pole during the light-on compared to the light-off treatment. Artificial light had a strong effect on bats at our study site, leading to a 50% increase in acoustic activity of migrating *P*. *nathusii* and *P*. *pygmaeus*. In the species group of nyctaloids, we documented a non-significant trend for an attraction towards artificial light, yet this species group includes both migratory species (*N*. *noctula* and *V*. *murinus*) and non-migratory species (*E*. *serotinus* and *E*. *nilsonii*), which might have hampered our ability to detect an effect. In addition, acoustic recording distances of the comparatively low-calling nyctaloid bat species (frequencies with most energy ranging between 20–30 kHz) may have been larger than the effective catchment area of the light treatment, i.e. we may have recorded nyctaloid bats that flew at a distance to the light treatment which was too large to affect these bats. Alternatively, artificial green light at night may only be affecting the migratory behavior of bats of the genus *Pipistrellus*. Further, even at 23 m distance to the central pole with the laser light, we found an increased activity for *P*. *nathusii* at the land-facing pole during the light-on treatment; yet, this effect was not present at the coastal pole where we observed a high activity of bats in general. Since the lateral poles were placed at a distance beyond the detection range of the bats’ echolocation calls for insects [42], we consider it most likely that the increase of *P*. *nathusii* individuals is indicative of positive phototaxis as such to artificial green light. The gradient of acoustical activity of bats from the sea-facing to the land-facing pole might be reflecting the fact that migratory bats use a small corridor at the shoreline and that the experimental site was only partly covering the center of this corridor.

We observed an overall low feeding activity of *P*. *nathusii* at our experimental site. At the central pole, we recorded only 2.5 FB per night. Therefore, migratory bats do not seem to consume large numbers of insects during migration flights. Since others and we observed migratory bats defecating shortly after they were captured at our study site [49], we speculate that migratory bats may hunt insects before they launch for long-distance migratory flights. We only observed a trend for a higher proportion of FB in relation to echolocation calls when the green light was switched on compared to darkness, suggesting that the feeding activity of *P*. *nathusii* did not increase disproportionately in relation to overall echolocation call activity when the light was switched on. Our observations suggest that bats did not hunt insects frequently at our site and that there was no disproportionate increase in feeding activity in relation to the overall echolocation call activity during the light-on treatment. Both findings are more consistent with the positive phototaxis hypothesis than with the attraction-by-insects hypothesis.

In summary, we conclude that migratory bats were not attracted to the light source because they responded to potential high insect densities at the light source, but because of the mere presence of green light. The specific reasons underlying the observed response behavior of migrating *P*. *nathusii* and other species towards green light remain unclear. Interference of artificial light with a magnetic sense seems unlikely in bats, since the molecule which is homologous to the avian Cry1a has no magnetoreceptive function in bats [50]. Instead, artificial light might interfere with the perception of celestial cues or with the use of other sensory modalities such as echolocation. For example, increasing light intensities seem to be in conflict with obstacle detection in bats, leading to higher risk of colliding with obstacles in illuminated habitats [51]. Alternatively, migratory bats may also inspect the novel stimulus presented to them in their flight trajectory.

## Conclusions

Our study is the first to show that migratory bats may respond with positive phototaxis towards green light at night, which demonstrates that migratory bats seem particularly susceptible to artificial light at night. Indeed, artificial light at night may represent a yet unrecognized anthropogenic stressor that possibly influences flight paths of individual migratory bats and that might interfere with bat migration in general. For example, it is well possible that the illumination of offshore platforms or buoys with green light could lure bats offshore where they may face an increased mortality risk compared to conspecifics migrating onshore. This is of particular concern, since migratory species are particularly vulnerable to other anthropogenic stressors as well, including for example the increasing numbers of wind turbines where large numbers of bats are killed worldwide [52–54].

## Supporting information

S1 Fig Location of the three experimental poles (black dots in the lefthand landscape picture). All poles (6.15 m height) carried an ultrasonic microphone (see schematic picture on the righthand side). The central pole was equipped in addition with a white board (8.9 m height in total) that was illuminated by green light (520 nm) in 10 min light-on light-off sequences. Lateral poles were at 23 m distance to the central pole.
(PNG)

S1 Table Data of Fig 1. Echolocation call activity of *Pipistrellus nathusii*, *P*. *pygmaeus* and nyctaloids at pole 0, 1 and 2.
(XLS)

S2 Table Data of Fig 2. Feeding buzz activity of *Pipistrellus nathusii*.
(XLS)
