# Supplementary figures and images for: Migratory bats respond to artificial green light with positive phototaxis
Source: PLoS One. 2017 May 31;12(5):e0177748. doi: 10.1371/journal.pone.0177748 (PMC5451015; doi:10.1371/journal.pone.0177748)

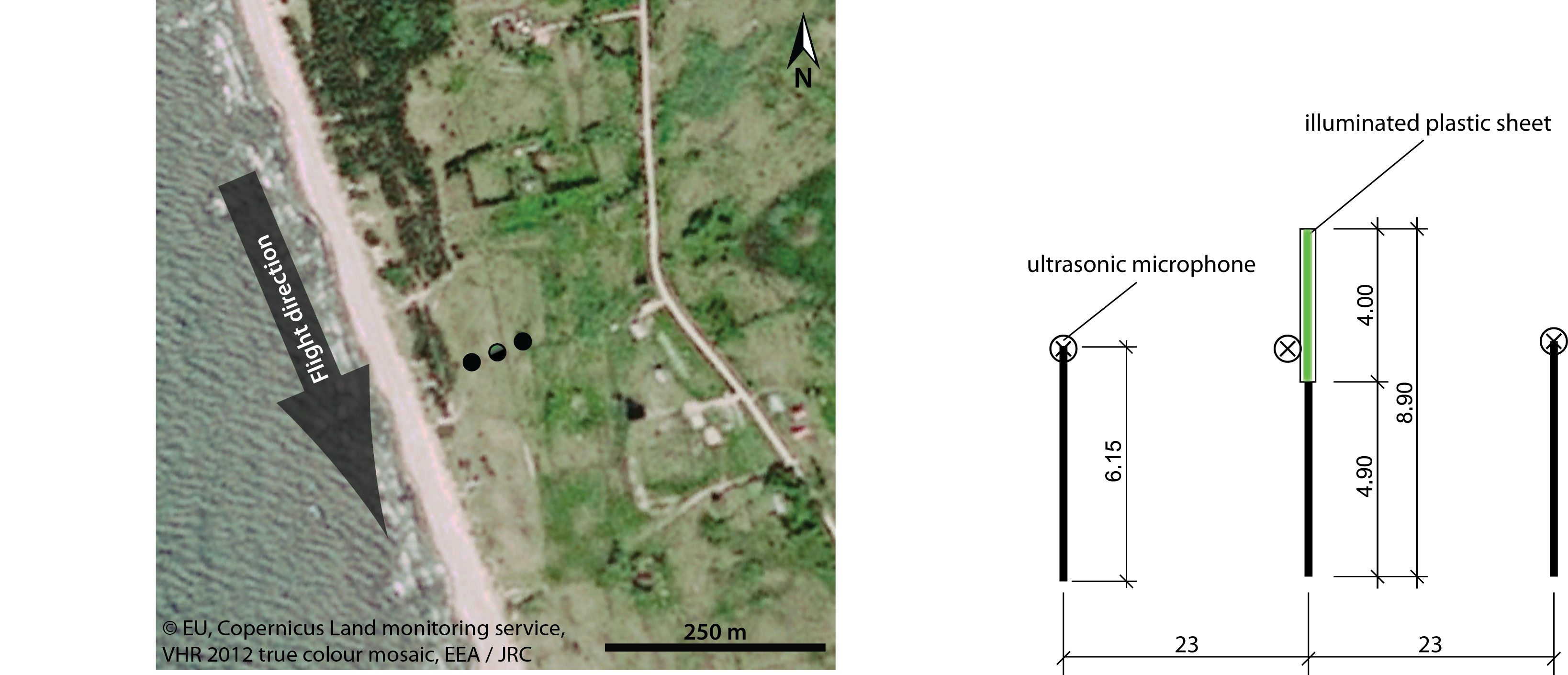

Supplement: S1 Fig — All poles (6.15 m height) carried an ultrasonic microphone (see schematic picture on the righthand side). The central pole was equipped in addition with a white board (8.9 m height in total) that was illuminated by green light (520 nm) in 10 min light-on light-off sequences. Lateral poles were at 23 m distance to the central pole. (PNG) [file pone.0177748.s001.png]
